# Messenger RNA vaccines for cancer immunotherapy: progress promotes promise

**DOI:** 10.1172/JCI156211

**Published:** 2022-03-15

**Authors:** Amanda L. Huff, Elizabeth M. Jaffee, Neeha Zaidi

**Affiliations:** 1 Department of Oncology,; 2 The Sidney Kimmel Comprehensive Cancer Center,; 3 The Skip Viragh Center for Pancreatic Cancer Research and Clinical Care,; 4 The Bloomberg-Kimmel Institute for Cancer Immunotherapy, and; 5 The Cancer Convergence Institute, Johns Hopkins University School of Medicine, Baltimore, Maryland, USA.

## Abstract

The COVID-19 pandemic has elevated mRNA vaccines to global recognition due to their unprecedented success rate in protecting against a deadly virus. This international success is underscored by the remarkable versatility, favorable immunogenicity, and overall safety of the mRNA platform in diverse populations. Although mRNA vaccines have been studied in preclinical models and patients with cancer for almost three decades, development has been slow. The recent technological advances responsible for the COVID-19 vaccines have potential implications for successfully adapting this vaccine platform for cancer therapeutics. Here we discuss the lessons learned along with the chemical, biologic, and immunologic adaptations needed to optimize mRNA technology to successfully treat cancers.

## Introduction

For decades cancer vaccines have been utilized to induce antitumor immune responses against cancer antigens, sometimes in association with clinical responses. Initial cancer vaccination strategies targeted antigens linked to oncogenesis and cancer progression that are shared by many cancers — either overexpressed cancer-initiating gene products (driver antigens) such as HER-2 or reactivated gene products such as MAGE (1, 2). With the advent of bioinformatics tools, studies now focus on vaccines targeting neoantigens personalized to individual patients. These studies have shown promise in activating robust and durable neoantigen-specific T cells, as well as early clinical responses, particularly in tumors such as melanoma that are more likely to respond to immune checkpoint blockade (3–7). Preclinical and early-phase clinical trials testing neoantigen vaccines in immunologically insensitive cancers, such as pancreatic ductal adenocarcinoma and glioblastoma, have also shown promise in inducing neoantigen-specific antitumor immunity (8–11).

While many studies have used DNA or peptide platforms for cancer vaccine delivery, mRNA-based therapeutics have demonstrated equal or greater activity in preclinical studies and early-stage clinical trials. The mRNA platform is also versatile and has successfully been used in systemic, subcutaneous, intramuscular, and in situ vaccine strategies and to genetically modify dendritic cell–based vaccines and create chimeric antigen receptor (CAR) T cell therapies. But progress in mRNA vaccine clinical development has been slow because of challenges relating to stability, cost of personalized production of patient-specific vaccines, and delivery. The SARS-CoV-2 pandemic led to the successful clinical development and application of several mRNA vaccines, underscoring the remarkable versatility, favorable immunogenicity, and overall safety of the mRNA platform on a global scale. A large part of the more recently conducted foundational research that led to this success has rested on transformative biomedical engineering and novel delivery methods aimed to optimize the therapeutic potential of this vaccine platform. Understanding the advancements that yielded successful mRNA vaccines for COVID-19 should accelerate progress in overcoming the remaining challenges for their application to cancer vaccination.

## History of mRNA-based vaccine strategies

As a novel expression platform, Malone and coworkers first documented the in vitro expression of a luciferase transgene from an mRNA vector (12). Shortly after, in 1990, Wolff et al. demonstrated the first successful RNA-mediated expression of a reporter transgene in vivo (13). In 1993, a liposome-mRNA construct expressing influenza hemagglutinin was reported to induce cytotoxic CD8^+^ T cell responses capable of detecting and lysing virus-infected cells in a murine model of influenza infection, demonstrating the immunogenic potential of RNA vectors (14). Two years later, vaccination with a naked mRNA encoding cancer embryonic antigen (CEA) induced CEA-specific antibodies in mice, underscoring the anticancer potential of mRNA therapeutics (15–17).

mRNA-based vaccines were first brought to the immunotherapy field for their attractive ability to safely vaccinate against proteins harboring oncogenic driver mutations. Notably, compared with DNA vectors, the mRNA platform provides robust antigen expression without necessitating transgene entry into the nucleus, thereby avoiding potential toxicity issues related to DNA integration. The rapid turnover rate of mRNA further eliminated concerns of potential off-target cell transformation events. Among the earliest constructs, replicative and non-replicative positive sense RNA virus genomes proved their amenability to modification, transgene expression, and immunogenic potential against viral and cancer antigens in several mouse models (18–20). In addition, a liposome-mRNA vaccine encoding human glycoprotein 100 (hgp100) was shown to activate cognate cytotoxic T cells and control tumor growth in a murine model of melanoma (21). In parallel, murine or human autologous dendritic cells transfected ex vivo with bulk tumor or single-antigen mRNAs were highly effective in priming antigen-specific T cell responses, thus broadening the scope of mRNA applications to adoptive cell transfer therapies (22–25). Such mRNA-based platforms promote antigen processing of endogenously translated antigens through the MHC class I antigen presentation pathway compared with conventional peptide pulsing that requires efficient cross-presentation to prime cytotoxic T cells. Collectively, this seminal work in cancer and infectious disease vaccine strategies has established mRNA as an immunogenic platform. The methodical modifications made to RNA vectors throughout their development have collectively led to substantial improvements in antigen expression and immunophenotype modulation, paving the way for broad vaccination applications against cancer and pathogen antigens (26–30).

## Lessons from global application of SARS-CoV-2 mRNA vaccines

The successful development, characterization, and application of mRNA-based SARS-CoV-2 vaccines along with the exceptionally high response rate to vaccination in diverse populations have propelled the urgent application of the mRNA platform in cancer treatment. As the first of their kind, the Pfizer/BioNTech (BNT162b2) and Moderna (mRNA-1273) SARS-CoV-2 mRNA–lipid nanoparticle (mRNA-LNP) vaccines established worldwide protocols for and feasibility of large-scale good manufacturing practice, broad vaccine distribution, and vaccine administration, from which several lessons were learned. Importantly, SARS-CoV-2 mRNA vaccine studies have uncovered the diversity of adaptive immune responses that are elicited and contribute to early and long-term disease control.

The open and rapid access to ongoing studies evaluating the immune mechanism of action of mRNA vaccines led to a considerable amount of new knowledge about mRNA vaccine responses in a short period of time. We learned that both vaccines are highly effective at inducing protective responses against SARS-CoV-2 infection in over 95% of individuals (31, 32). This was much higher than was predicted based on knowledge of similar responses associated with other vaccine platforms such as live attenuated vaccines. Polyclonal, neutralizing IgG antibodies against the spike protein were detectable at high levels 3 to 4 weeks after initial vaccination (33–35). Further, both vaccines induced Th1-type CD4^+^ T cells and cytotoxic CD8^+^ T cell responses (33, 36, 37). Phenotypically, the polyclonal CD8^+^ T cells generated were found to be early differentiated effector cells at day 85 after vaccination (33). Durable antibody and antigen-specific memory T cells have been detected 6 to 7 months after vaccination (35, 38, 39). The observed differences in protective efficacy between these vaccines likely underscore the immunologic effects of vaccine dose, minor variations in the respective mRNA vector constructs, or lipid-based delivery (33, 34, 37, 40, 41). In this regard, a third mRNA vaccine tested, CureVac, comprising an unmodified mRNA vector, demonstrated poor immune responses in phase III trials, which is attributed to the lower dose given and/or absence of chemical modifications to promote antigen expression.

A major challenge in the application and distribution of these mRNA vaccines is the required storage and delivery at –80°C, and the relatively short shelf life of approximately 6 months (42–44). Upon dilution, their half-life is also short, only 6 to 12 hours at room temperature or 5 to 30 days at 4°C. This challenge necessitates improvement in product longevity. Several groups have attempted to increase nanoparticle-RNA stability through various formulations of lipid polyplexes, lipoplexes, and lipopolyplexes (45–47). The development of a lyophilized mRNA-LNP should further ease delivery and storage requirements (43).

While the SARS-CoV-2 mRNA vaccines showed favorable safety profiles across diverse populations, side effects after intramuscular delivery of 30 μg of Pfizer’s mRNA construct, BNT162b2, were mild to moderate, and included pain at the injection site, fatigue, fever, chills, and headache (32, 48). Moderna’s mRNA platform, mRNA-1273, given at 100 μg, had similar side effects (31, 34, 49). Low-frequency anaphylaxis is also suspected to arise due to the inclusion of PEG, a component of the nanoparticle, although this remains to be established (50–52). These serious adverse events are rare, can be managed, and should not prohibit further development of this vaccine platform for other indications.

Finally, a major factor in the success of the SARS-CoV-2 mRNA vaccines was the commitment by pharmaceutical companies to rapidly manufacture vaccine candidates. Noting that many companies are now poised for large-scale production of flexible vaccine platforms, they are also positioned to develop novel cancer vaccine candidates much more rapidly. To aid in this effort, continued development of constructs with improved uptake, stability, and potency should ease production costs by reducing dose requirements and improve therapeutic indexes to successfully employ mRNA vaccines for cancer treatment.

## Immunologic requirements for mRNA vaccines to treat cancer

Most antigen delivery systems are difficult to rapidly engineer, modify, or produce, lack potent immunogenicity, and/or cause neutralizing immunity to the delivery system itself. However, transformative advances in bioengineering, rapid manufacturability, and immunogenicity of the mRNA platform provide a unique opportunity to target a broad range of antigens for cancer vaccination. Nonetheless, there are a set of unique immunologic requirements for successful vaccine strategies against cancer-associated immunogens, particularly when considering prevention, immediate control of existing disease, and long-term protective immunity (53). Vaccination to prevent infection with pathogens requires robust production of antibodies by B cells, aided by CD4^+^ T cell help, to neutralize pathogens upon initial infection and reinfection (37, 54). Further, it is suspected that low levels of cytotoxic CD8^+^ T cells contribute to early control of viral infections before sufficient antibody responses are mounted (38, 55, 56). In contrast, treatment and prevention of most cancers require robust and diverse cytotoxic CD8^+^ T cells to directly debulk primary or metastatic tumor burdens, which are composed of heterogeneous tumor cells expressing many tumor antigens. A diverse T cell repertoire is required to meet the changing antigen landscape as cancers evolve to escape immune recognition (57). Furthermore, CD4^+^ T cells are increasingly recognized as both direct and indirect contributors to immunotherapeutic efficacy (3, 4, 58, 59). Importantly, CD4^+^ Th1 cells have been preclinically and clinically implicated in maintaining control of tumor growth, even in tumor models that lack MHC class II expression (59–64). mRNA vaccines are among the few antigen delivery systems with the potential to simultaneously deliver multiple antigens that can activate potent and diverse CD4^+^ and CD8^+^ T cell responses.

mRNAs are immunogenic delivery systems in part because they naturally target antigen-presenting cells (APCs) — notably macrophages and dendritic cells — the specialized immune cells that most effectively prime T and B cells (65). Thus far, mRNA vaccination strategies for cancer treatment have shown success in murine cancer models and in patients targeting an array of tumor antigen types, including the tumor-associated antigens hTERT, Melan-A, gp100, tyrosinase, WT-1, and PRAME; the tumor-specific antigens CEA, MUC1, survivin, p53, NY-ESO, MAGE-A1, MAGE-A3, and CMV-pp65; oncoviral proteins; patient-specific neoantigens; or CAR T cell targets. Despite these early successes, the cost, scalability, and delivery issues have historically contributed to their slow adaptation for cancer treatment.

An additional challenge for cancer vaccines in general is overcoming the local and systemic immunosuppressive mechanisms often associated with transformed cells that prevent priming or effector function of anticancer T cells. While most tested vaccines demonstrate priming of antigen-specific T cell responses, the immunosuppressive tumor microenvironment (TME) of cancers often hinders T cell access and function at the tumor site. Immune checkpoint therapies successfully reprogram one or more immunosuppressive signals in the TME to allow T cell access and function. Thus, in situ vaccines that successfully induce anticancer T cell responses require the co-delivery of costimulatory molecules or immune checkpoint inhibitors to achieve antitumor immunity (66). Because mRNA vaccines are easy to engineer and offer flexible delivery options, it is possible to deliver mRNA vaccines encoding tumor antigens along with or in parallel with diverse immunomodulatory agents. Interestingly, mRNA vaccines have also been engineered to target and induce immunogenic cell death of tumor cells directly (67). The success of SARS-CoV-2 mRNA vaccines has provided new opportunities for application to cancer therapeutics. Below, we discuss the biomedical engineering of mRNA vectors and liposome delivery systems that have the potential to promote anticancer responses through these pathways.

## Biologic and immunologic qualities underpinning mRNA vaccine immunogenicity

There are three broad categories of mRNA vaccine constructs: non-replicating, self-replicating, and trans-replicating (or splitzicon) RNAs. Non-replicating and self-replicating RNAs have been primarily used in cancer vaccine strategies. Non-replicating mRNAs, such as the SARS-CoV-2 vaccine, encode the target antigen that is translated into protein immediately after uptake into the target cell’s cytoplasm. This results in initial high protein expression that diminishes over a few days (68). The production of non-replicating mRNA constructs is relatively simple through in vitro transcription or the isolation of whole-tumor transcripts. In contrast, self-replicating constructs contain not only the transgene of interest, but also the viral RNA-dependent RNA polymerase (RdRp) that amplifies the viral genome. These latter vectors not only produce greater amounts of protein, but also induce stronger innate signaling due to sensing of double-stranded RNA intermediates (69). This strategy is potentially most attractive for cancer vaccines, providing higher quantities of immunogen and strong acute inflammatory signals, both required for inducing durable antigen-specific adaptive responses. Finally, trans-replicating, or splitzicon, RNAs deliver the transgene of interest and the viral RdRp on separate transcripts to reduce the size of the RNA vectors needing to be encapsulated during production (70–72). This additional feature may be helpful in developing mRNA vaccines that deliver multiple antigens for immunization.

**Figure 1:**
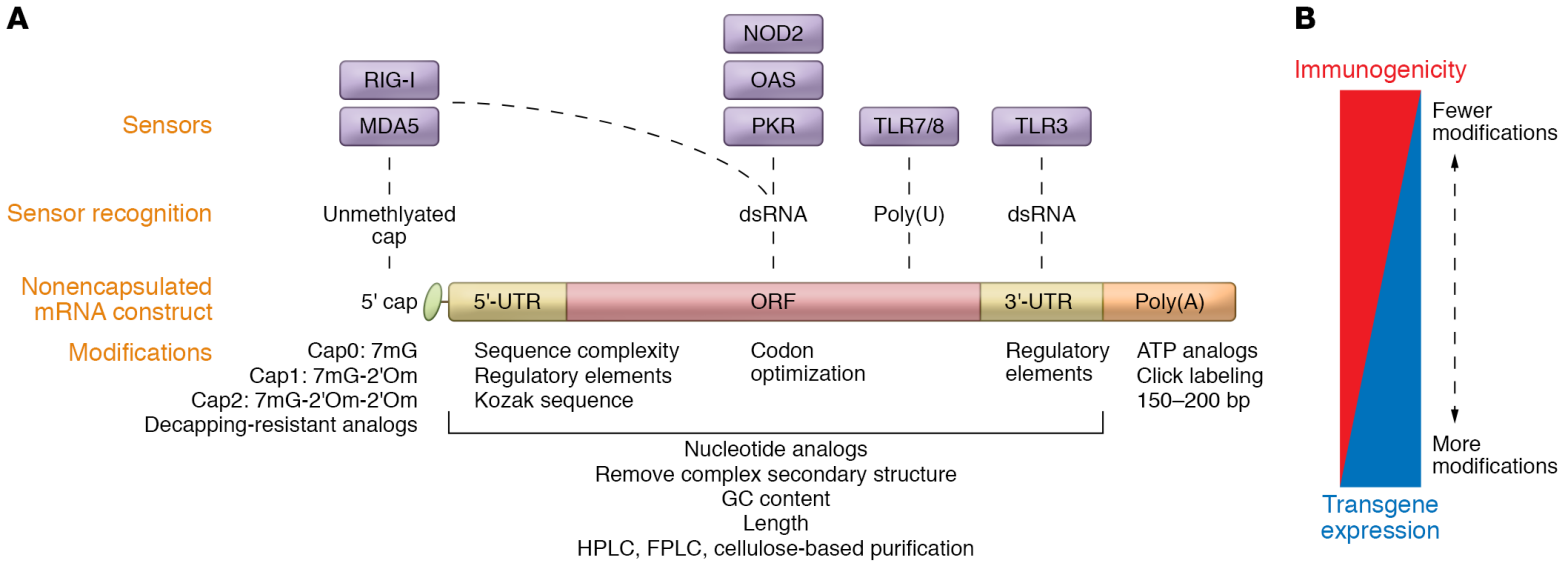
Modifications of mRNA vaccines for enhanced antitumor immunity. Several strategies have been used to chemically modify mRNA constructs to optimally balance antigen expression with innate immune recognition of the mRNA construct itself. (**A**) Manipulations that enhance antigen expression include modification in the 5′ cap through anti-reverse cap analogs, methylation of start nucleotides, and decapping-resistant analogs; modification in untranslated regions through the removal of long stem-loop-like structures with high GC content, insertion of an internal ribosomal entry site within the 5′-UTR, and inclusion of a Kozak sequence upstream of the start codon; modification in the open reading frame (ORF) through codon optimization based on target cell tRNA abundance; and modification of the poly(A) tail by incorporation of ATP analogs and click-labeling with fluorescent dyes. The ideal polyA length within human cells is approximately 120 bases. Use of nucleotide analogs, sequence complexity, GC content, and length modifications can reduce detection by innate sensors such as TLR7/8 or TLR3 or cytoplasmic sensors such as RIG-I, MDA5, OAS, NOD2, and PKR. Purification of in vitro–transcribed RNAs by HPLC, FPLC, or cellulose-based methods can further remove contaminating dsRNA products that would engage these sensors. (**B**) While these alterations result in greater transgene expression and less immunogenicity to the mRNA construct, the optimal combination of modifications and balance of the two to yield a therapeutic advantage is still an open question as it relates to optimization of cancer vaccines.

A key consideration in developing mRNA vaccines for cancer treatment is the optimal balance of innate sensing of the mRNA construct and vector expression. Exogenous mRNAs are inherently immunogenic, as their features are detected by several cellular pattern recognition receptors (PRRs) (Figure 1). Collectively, these innate signals result in the shutdown of RNA translation as well as degradation mechanisms that limit expression of the therapeutic payload. Among these, Toll-like receptor 3 (TLR3) recognizes doubled-stranded RNA in the endosomal compartment and signals through TRIF and IRF3, resulting in type I interferon production. Unmodified guanosine- and uridine-rich single-stranded RNA segments are recognized by TLR7 and TLR8 (73). Further, several cytosolic innate immune sensors, such as RIG-I, MDA5, and OAS, detect double-stranded RNA of foreign intracellular RNAs. Recent experience with mRNA vaccines has yielded multiple broad strategies to achieve this balance through modifications that reduce innate sensing or improve transcript stability and maximize translation. In the following sections, we discuss the modifications that have been methodically investigated to balance innate immune detection and transgene expression.

## Chemical modifications balance mRNA immunogenicity with antigen expression

To mitigate innate sensing of the chemical RNA backbone, incorporation of nucleoside analogs such as pseudouridine or *N*^1^-methyl-pseudouridine into the mRNA construct during in vitro transcription, as was used in both SARS-CoV-2 mRNA vaccines, dramatically reduces TLR recognition (28, 29, 74–77). Sequence engineering to increase guanosine-cytosine (GC) content can lessen innate immune sensing of RNA (78). Additionally, the removal of double-stranded RNA structures improves protein translation and reduces mRNA degradation (79–81). An alternative strategy is to augment STING-mediated innate sensing to bypass TLR signaling through heterocyclic lipid carriers. In both melanoma and HPV-associated murine tumor models, this strategy lowered systemic cytokine expression after sensing of the mRNA backbone, while maintaining innate signaling involved in enhancing antigen processing and presentation and reducing tumor growth (82). Moreover, co-delivery of constitutively active PRRs, such as a mutant STING, enhanced activation of vaccine antigen target HPV E7–specific CD8^+^ effector memory T cells in mice, leading to improved control of tumor growth (83). Importantly, the magnitude and kinetics of type I interferon responses induced by mRNA vaccines have been shown to have both beneficial and detrimental effects on mRNA-mediated induction of cytotoxic T cell responses (84). Thus, the balance between strong inflammatory signals provided by the vaccine and efficient transgene production is still being defined for cancer vaccine development (Figure 1).

Several mechanisms have been used to enhance transgene expression and selectively reduce innate detection that aims to eliminate the mRNA vector. One such approach has been through modification of the 5′ cap structure of the mRNA transcript, as has been achieved for both SARS-CoV-2 vaccines. RNA caps play a critical role in transcript stability and in distinguishing self-RNAs from foreign RNAs (85–88). An array of anti-reverse cap analogs (or ARCAs) have been developed and used in clinical products to generate mRNA constructs with enhanced antigen expression (89–91). Building on this technology, the CleanCap (TriLink Biochechnologies) method, used in the production of mRNA-1273, generates the cap1 structure, which includes methylation of the first transcribed nucleotide, a strategy that further improves translation efficiency. As the removal of the cap, or decapping, initiates transcript degradation (88, 92, 93), there has been a surge of interest in developing decapping-resistant cap analogs that improve transcript stability and enhance target antigen expression (94–98).

In addition, an altered 5′ cap structure can result in modified affinities for translational initiation factors, such as eIF3E, yielding a therapeutic advantage (99, 100). Notably, the dysregulation of translation initiation factors is a hallmark of cancer cell transformation. Recent studies have shown that methylation of adenosine start nucleotides using synthetic *S*-adenosyl-L-methionine analogs significantly improves protein expression and vaccine immunogenicity in in vitro systems (101). Understanding the role of cap binding proteins in transcript translation within cancer cells or immune cells should further inform the rational design of mRNA vectors with improved stability and translation efficiency.

Enhanced antigen expression can also be achieved by alteration of the 3′- and 5′-UTRs of the mRNA construct, including its secondary elements that are central for transcript stability and translation efficiency (102–104). These maneuvers include removing long stem-loop-like structures with high GC content (105, 106); inserting an internal ribosomal entry site within the 5′-UTR (107); and including a Kozak sequence (GCCACC) upstream of the start codon (108). In hypoxic conditions, such as within the TME, an initiation complex composed of eIF2E3, HIF-2α, and RBM4 binds to hypoxia response elements present in specific 5′-UTRs to promote translation. Predictive algorithms of 5′-UTR translation efficiencies should lead the way for the development of novel constructs through deep learning with improved transgene expression profiles (109). mRNA constructs with longer 3′-UTR regions have longer half-lives (110) and greater transcript stability (111). Transcript half-life can also be improved by removal of miRNA-targeting sites and AU-rich regions (112). Moreover, use of tandem β-globin 3′-UTRs has been shown to significantly improve transcript stability and antigen expression (4, 113).

Furthermore, it is critical to avoid highly rigid secondary structures in the open reading frame of an mRNA construct, which may slow ribosomal scanning, leading to RNA decay (114, 115). Several computational algorithms can predict transcript stability based on sequence structure (116, 117), one of which not only predicts RNA structure, but also indicates modifications that would prevent hydrolytic degradation (118). Codon optimization based on transfer RNA (tRNA) abundance can also improve translational efficiency. As tRNA abundance is cell type–specific and is dysregulated in cancer cells, mRNA vectors should be appropriately codon optimized based on the target cell of interest (119). Notably, cells within the spleen and lymph nodes have the highest abundance of tRNA relative to other tissues, suggesting that codon optimization may be less important for immune cell targets (120). Conversely, some proteins require slower translation rates for proper folding to occur, and thus codon optimization may not be ideal.

Finally, another chemical modification to improve RNA stability is to alter the poly(A) tail by incorporating ATP analogs, such as ATPαS, with non-bridging atoms at the α-phosphate; this stabilizes the transcript without impacting translation (121). Similarly, click-labeling at the poly(A) tail with fluorescent dyes increases translation (122, 123).

## Designing mRNA vaccines to optimize antitumor immunity

**Figure 2:**
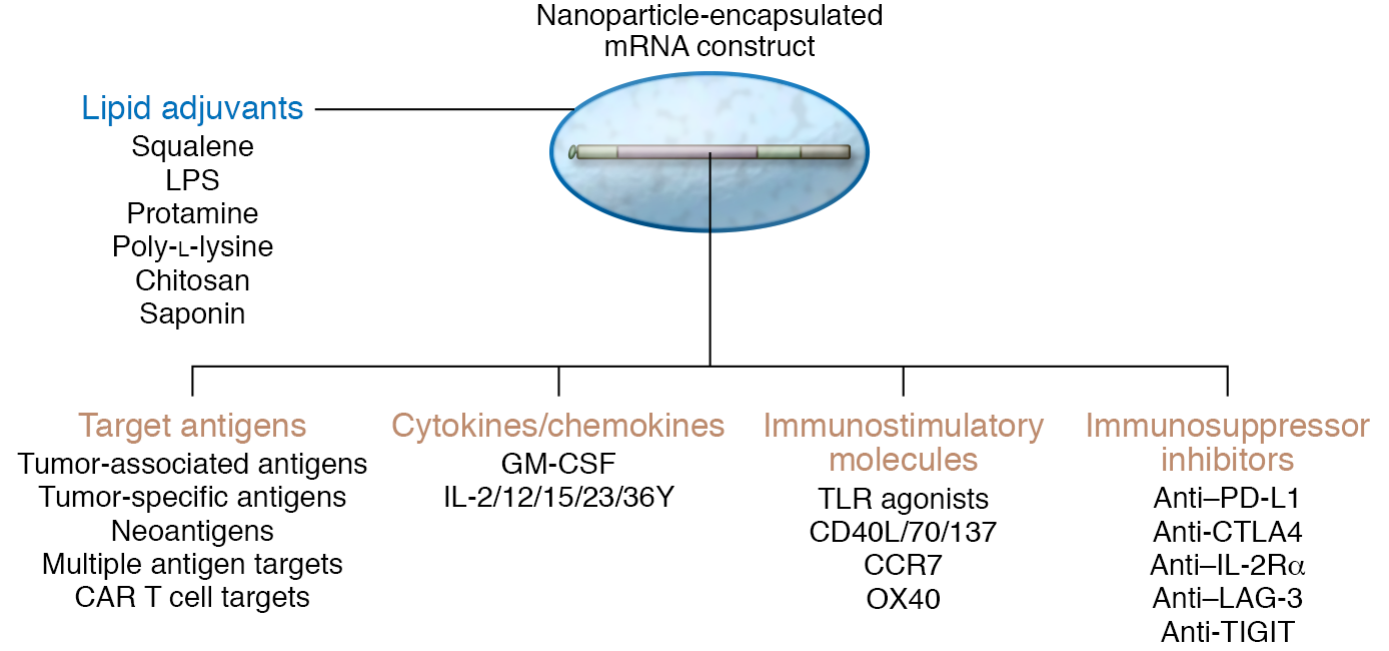
Enhancing the adaptive response of RNA vaccines for anticancer therapy. The induction of a robust and durable adaptive antitumor immune response with mRNA vaccines can be enhanced in several ways, highlighting the flexibility of the platform. These include delivery of lipid adjuvants within the nanoparticle; targeting multiple target antigen types, including those that are tumor–associated and tumor–specific antigens, such as neoantigens, and CAR T cell targets; employing cytokines or chemokines; and encoding or co-delivering immunostimulatory molecules and immunosuppressive inhibitors. These strategies should not only induce high-quality antigen-specific T cells, but also reprogram the tumor microenvironment in favor of a robust and durable anticancer response.

Cancer cells develop multiple mechanisms that modulate both local and systemic immunosuppression, including metabolic reprogramming, recruitment of immunosuppressive cells, and upregulation of inhibitory cytokines and signals. For successful treatment outcomes with mRNA-based therapeutics, the vaccine should ideally induce high-quality antigen-specific T cells and simultaneously reduce immunosuppressive signals within the TME. In this regard, modulation of the cytokine milieu in which T cells engage cognate antigen influences cell fate and memory phenotype. In addition to robust cytotoxic effector cells, generation of stem-like, memory T cells is important for durable immunologic memory and cell renewal in cancer therapy (124, 125). One advantage of an mRNA vector is the relative ease of co-delivering cytokines, immunostimulatory or suppressive cell type–depleting molecules that, in essence, address these distinct requirements for successful generation of robust and durable anticancer T cells (Figure 2). This strategy requires identifying and including the most potent vaccine adjuvant, as well ensuring the delivery of key immunomodulatory drugs either within the same mRNA vector or in a trans-replicating (splitzicon) construct, as discussed above.

Multifunctional innate immunoadjuvants have been co-delivered, including natural lipids, such as squalene, LPS, and saponin; synthetic lipids; polymers, including protamine, chitosan, and dextran sulfate; and synthetic polymers, such as polyethylenimine and poly-l-lysine (126). However, although co-delivery of certain non-lipid-like adjuvants with mRNA vaccines requires careful chemical packaging, preclinical studies with gardiquimod, a TLR7 agonist, in poly(lactic-*co*-glycolic acid) (PLGA)–core/shell RNA-nanoparticle–induced potent antitumor immune responses (127).

mRNA-based vaccines provide a platform for co-delivering a range of immunomodulatory agents, thus allowing a fine-tuning of T cell responses. For example, co-delivery of stimulatory cytokines, such as GM-CSF, IL-12, and IL-15, with mRNA encoding tumor antigen in dendritic cell vaccines promotes remodeling of the TME, enhances cytotoxic T cell responses, and controls tumor growth in preclinical models (128–131). Further, in situ vaccination with a cocktail of naked mRNAs encoding IL-12, GM-CSF, IL-15, and IFN-α4 promotes tumor infiltration of polyfunctional Th1-like CD4^+^ cells, cytotoxic CD8^+^ T cells, and pro-immune monocytic cell types while decreasing Tregs, leading to survival benefits in murine models of cancer including metastatic melanoma (132). Similarly, in melanoma patients, coexpression of immunostimulatory molecules, such as CD40L, CD70, and constitutively active TLR4 (TriMix, eTheRNA Immunotherapies), along with the tumor antigen mRNA potentiates APC maturation and cognate T cell activation (133, 134). Several studies have also documented the benefit of mRNA-based vaccination against a variety of tumor antigens in combination with immune checkpoint blockade, such as anti–PD-1 and anti-CTLA4 targeting antibodies in both murine and human therapy studies (4, 135). In this regard, it has been shown that mRNA delivery of TYRP2 plus siRNA against PD-L1 reduces expression of PD-L1 on dendritic cells, increases cognate T cell activation and proliferation, and controls tumor growth in murine melanoma (136). Likewise, depletion of immunosuppressive Treg populations and myeloid-derived suppressor cells by chemotherapeutics or depleting antibodies delivered along with antigen-encoding mRNAs slowed tumor growth (137). Finally, the potential to broadly apply the mRNA platform in diverse cancer immunotherapy strategies as an immune activator is underscored by its use in adoptive cell therapies for both ex vivo generation and in vivo expansion of CAR T cells by encoding of CAR constructs or CAR T cell targets, respectively (138, 139).

## Targeting mRNA vaccines to immune and cancer cells

Focused approaches to enhance mRNA vaccine uptake into immune or cancer cells are under way with a focus on improving therapeutic index and limiting off-target toxicity (140, 141). Ex vivo transfection of autologous dendritic cells constituted the earliest attempts to ensure the delivery of mRNA-transfected APCs to patients. Preconditioning of human APCs with cytokines, such as TNF-α, or TLR agonists was shown to improve dendritic cell trafficking into the lymph nodes and enrich presentation of transfected antigen to T cell populations (142–144). However, the expense and labor-intensive protocols associated with delivering reproducible biologic product limit broad applicability.

Nonencapsulated mRNA vaccines degrade rapidly within 5 minutes when injected systemically (145), while widely used liposome encapsulation has resulted in a marked improvement in stability and in vivo targeting, thus broadening the scope of injection routes for mRNA delivery (146, 147). Lipid nanoparticles are generally composed of four components: ionizable lipid that dictates particle charge, cholesterol, a helper lipid, and PEGylated lipids that aid in particle stability, formation, and membrane-membrane fusion in the cell (45, 148, 149). The size, shape, and surface charge influence biodistribution and cell-type uptake (150, 151). Cationic lipids, including DOTMA and DOTAP, have most commonly been used to encapsulate anionic RNAs efficiently. Particle size also directly correlated to biodistribution. Lipoplexes around 200 to 400 nm in size accumulate preferentially in the spleen after intravenous delivery (150, 152), while smaller particles, optimally 20 to 50 nm, are required for trafficking into lymph nodes (153–155). Modification of lipid complexes to display mannose domains has been shown to improve dendritic cell uptake, antigen expression, and antitumor responses in murine melanoma models (156). Importantly, dendritic cell populations including plasmacytoid, classical type 1, and classical type 2 have distinct capabilities to process and present antigen, utilize cross-presentation pathways, and produce inflammatory signals. Thus, targeting these cell types individually promotes differential cognate T cell responses with classical type 1 dendritic cells being the primary target for cancer vaccines thus far given their superior cross-presentation capability (157–159). A recently developed mRNA hydrogel vaccine containing the TLR7/8 adjuvant R848 for the treatment of metastatic melanoma increased dendritic cell uptake of the mRNA vaccine, significantly enhanced CD8^+^ T cell responses, and provided durable antitumor immunity in preclinical models (160). The SARS-CoV-2 mRNA vaccines used Lipid H (Cayman Chemical, SM102) or Acuitas ALC-0315 ionizable lipids in mRNA-1273 and BNT162b2, respectively (161, 162).

Targeting tumor cells directly to deliver immunogenic payloads or to induce oncolysis provides an alternative mechanism to reduce tumor burden and initiate remodeling of the TME. Leaky tumor vasculature and damaged lymphatic structures within the TME improve retention of systemically delivered nanoparticle constructs. Thus, similar to immune cell targeting, particle size and charge dictate propensity for tumor cell–specific uptake (150). It was recently shown that manipulation of the particle lipid content can promote tumor cell uptake and cell death (67). Notably, *N*^1^,*N*^3^,*N*^5^-tris(2-aminoethyl) benzene-1,3,5-tricarboxamide–formulated (TT-formulated) nanoparticles promoted particle uptake in tumor cells in vitro and in vivo, and induced cell lysis while conferring IL-12 transgene expression (67, 163, 164). These cytotoxic tumor-targeting particles induced robust and durable CD8^+^ T cell–mediated reduction of tumor growth in a murine B16F10 melanoma model (67, 164). Alternatively, mRNAs have been used to reintroduce tumor suppressors such as TP53 and PTEN to tumors, sensitizing them to chemotherapeutics and delaying tumor growth in preclinical models (145, 165). A number of tumor-targeting constructs also rely on hypoxic conditions, selective antigen expression, and antibody display to promote tumor cell uptake; however, the use of these particles for mRNA delivery has been limited. A recent report of successful selective organ-specific targeting with mRNA delivery provides a potential opportunity to enhance tumor targeting based on tissue location (166). Regardless of the cell type targeted, mRNA delivered in nanoparticle carriers needs to undergo endosomal release into the cytosol for translation to occur. Endosomal release is largely modulated by the p*K*_a_ of the particle, which is directly related to ionizable lipid shape and size (167, 168). Many endosomal release methods have been characterized; however, they have not been broadly compared (167, 169–171).

Intradermal, subcutaneous, and intramuscular injection routes of any mRNA vaccine capitalize on the presence of diverse APC populations within the skin and muscle (172, 173). Notably, HIV antigen–containing lipid nanoparticles delivered by intramuscular and subcutaneous routes displayed distinct anatomical lymph node trafficking and sites of T cell activation (174), but without differences in T cell responses (174). Intramuscular injection, however, resulted in the longest sustained antigen expression over time that is directly related to mRNA half-life (68) — thus, both SARS-CoV-2 mRNA vaccines, BNT162b2 and mRNA-1273, are delivered intramuscularly. In contrast, intravenous delivery induces more robust cytotoxic T cell responses (152, 175), but with greater particle uptake in the liver and possible off-target toxicity, requiring a more careful design of the targeting nanoparticles for successful delivery by this route.

## Approaches to test mRNA cancer vaccines in humans

mRNA vaccines have been studied in different formats and settings and as part of combinatorial therapies in cancer clinical trials (Table 1). Two strategies that have shown promise include dendritic cell–based mRNA pulsing and lipid-complexed mRNA vaccines. An early approach had been to load dendritic cells with cancer antigens by pulsing with mRNA that was mostly generated by in vitro transcription or obtained from autologous cancers or cancer stem cells. An example of this approach was a phase I trial that pulsed autologous Langerhans-type dendritic cells with xenogeneic TRP-2 mRNA. Stage IIB to IV melanoma patients who had their tumors resected were given five vaccines every 2 weeks. Six of nine patients remained disease free for a median of 51.1 months, and generated robust immune responses, including T cell activation, cytokine release, and increased clonality, with minimal signs of toxicity (176). A major drawback that prevented widespread testing was the inability to develop these somewhat cumbersome biologic agents at a reliable quality and quantity on a large scale.

A second approach using lipid-complexed mRNAs demonstrated both favorable safety and robust immune responses against immunizing antigens expressed by different tumor types (Table 1). For example, patients with metastatic gastric, colon, and rectal cancers were vaccinated against up to 20 shared and personalized antigens delivered in a single lipid-complexed mRNA construct (named mRNA-4650). The vaccine was safe and induced neoantigen-specific T cells, but without objective clinical responses (177). This reinforces the need to combine these vaccines with immune modulation to achieve clinical benefit in immune-insensitive tumors. In contrast, in patients with melanoma, an immune-sensitive cancer, an interim analysis of an ongoing first-in-human dose escalation phase I trial has documented efficacy of an intravenously administered liposomal RNA vaccine, FixVac (BioNTech, BNT111), which targets four melanoma-associated antigens. Notable, objective responses were observed in patients who had previously received checkpoint blockade, and these responses correlated with the induction of de novo vaccine carrying neoantigen-specific CD4^+^ and CD8^+^ T cells (5).

mRNA vaccines can also be enhanced using TriMix, a cocktail of three naked mRNAs encoding constitutively active TLR4 to facilitate dendritic cell antigen presentation, and CD70 and CD40L to activate CD8^+^ and CD4^+^ T cells, respectively (178). Both the naked TriMix mRNA and ex vivo dendritic cell–loaded TriMix mRNA have shown efficacy in multiple preclinical and clinical studies, mainly through increased dendritic cell activation and by shifting the CD4^+^ T cell phenotype from Tregs to Th1-like cells (134, 179, 180). Notably, 27% of patients with stage III or stage IV melanoma who were immunized using dendritic cells loaded with mRNA encoding the melanoma-associated antigens MAGE-A3, MAGE-C2, tyrosinase, gp100, and TriMix showed tumor regressions (179).

Human studies testing mRNA vaccines in cancer have provided evidence for safety and immunogenicity. However, these trials have had a relatively small sample size, with clinical responses that are mixed, despite robust immune responses seen peripherally. This is particularly true in patients with “immunologically cold” tumors, such as glioblastoma. These early studies reiterate the need for co-delivery of immunomodulating agents to effectively bypass tumor-specific immunosuppressive signals. Furthermore, most of the immune responses are evaluated using assays requiring ex vivo expansion, which provide an antigen-specific readout but may not accurately quantify and assess the quality of the overall T cell response in vivo.

## Steps to advance mRNA vaccines for cancer therapeutics

**Figure 3:**
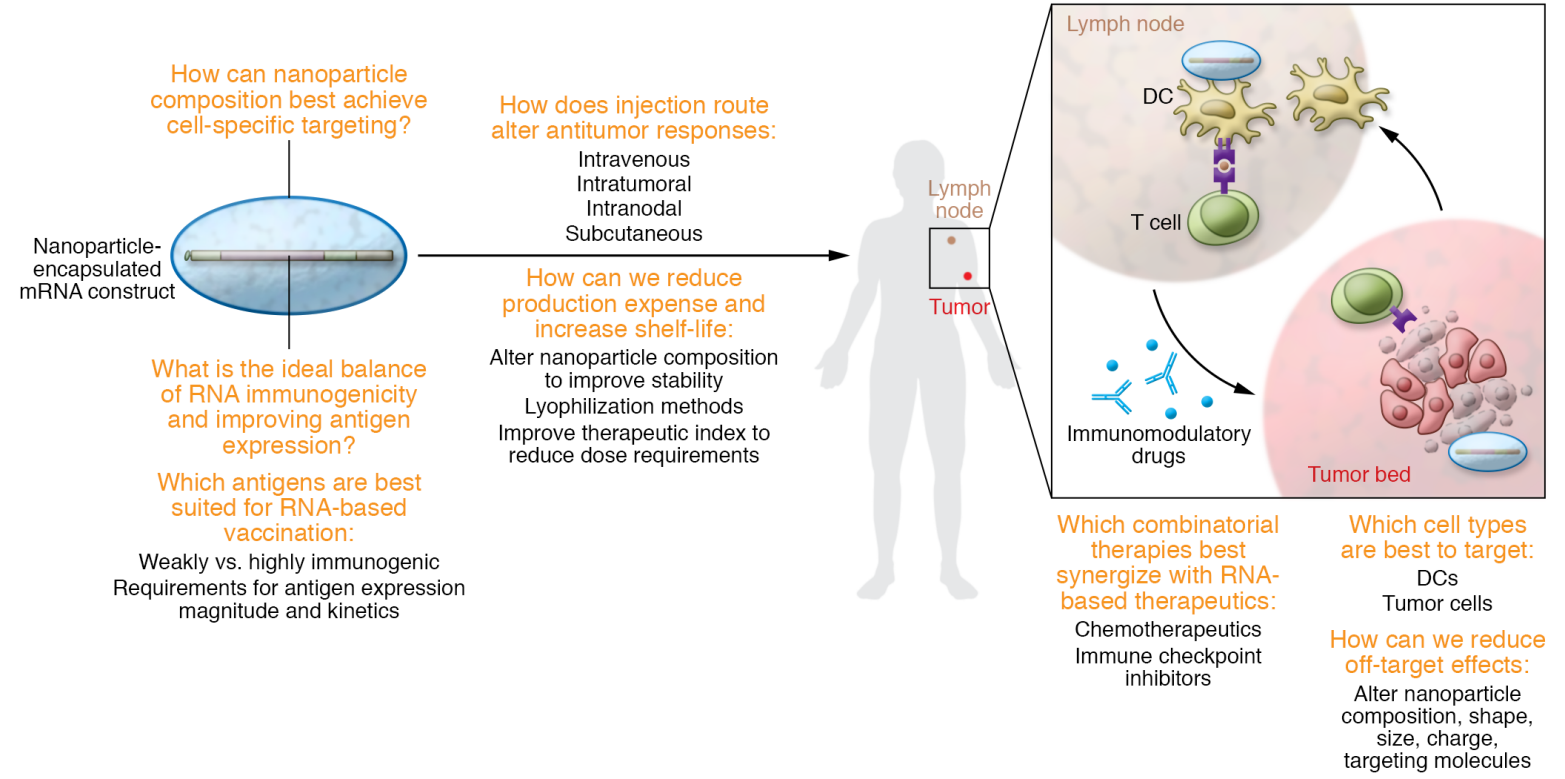
Outstanding questions for RNA-based cancer therapeutics. Several questions remain as to how mRNA vaccines can be best applied for cancer treatment. These include questions related to optimal cell-specific targeting; balancing of antigen expression with immunogenicity of the mRNA construct; optimal cancer antigens to be targeted; route of injection; manufacturability and stability of the vaccine; minimizing of off-target effects; and optimization of combinatorial therapies to synergize with mRNA vaccines.

Early mRNA-based therapeutics have shown promise in cancer therapy. Clinically, these agents can be potent T cell inducers and can also remodel the immunosuppressive TME. However, because of the inherent heterogeneity of cancer both within and across clinical indications, additional technological advances are needed to develop successful RNA-based cancer therapies (Figure 3). Ongoing chemical engineering initiatives are adapting mRNA vectors to optimize the balance between antigen expression and innate immune sensing of the vector to achieve a therapeutic advantage (Figure 1). Furthermore, the tumor antigen target type may require different levels of immunostimulatory signaling to overcome systemic or local tolerance. The ideal combination of antigen and immunomodulatory targets included in the vaccine construct needs to be defined and is likely cancer type specific or even patient specific (Figure 2). There has been good progress in developing lipid nanoparticle carriers to improve mRNA platform stability, pharmacology, and target cell specificity. Since particle size, charge, and shape have direct implications for pharmacokinetics and pharmacodynamics, it is critical to define the immunologic benefits and disadvantages of different delivery routes to optimize anticancer immune responses. A critical question remains as to which immune cells should be the target and how best to deliver the mRNA to achieve the optimal therapeutic benefit, while limiting off-target toxicity. Lastly, the development and global distribution of the COVID-19 mRNA vaccine have underscored the feasibility and safety of mRNA-based vaccination in diverse groups worldwide. The now proven ability to manufacture these agents rapidly and efficiently has signified a giant step forward for future cancer vaccination applications. However, continued efforts are required to improve vaccine stability, and to reduce the cost of these therapeutics, particularly if this approach is to be used in precision-based, patient-specific treatments.

In summary, considerable progress has been made in mRNA-based cancer therapeutics. This progress is made more tangible with the global success of the SARS-CoV-2 mRNA vaccines, further promoting promise for cancer immunotherapy.

## Figures and Tables

**Table 1:**
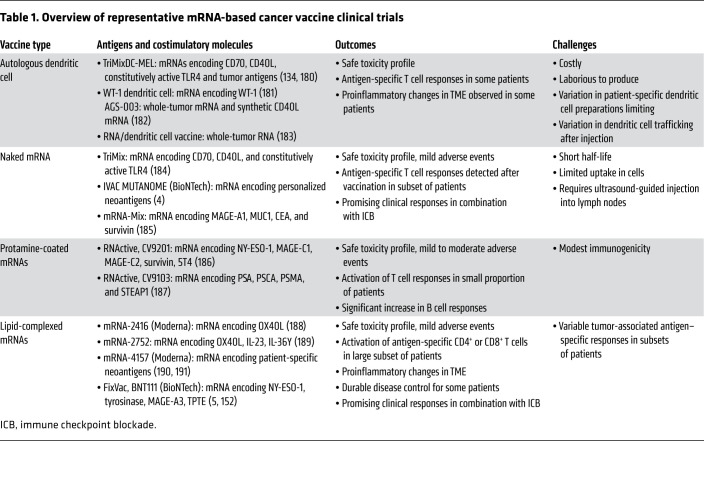
Overview of representative mRNA-based cancer vaccine clinical trials

## Version 1 03/15/2022

electronic version

## References

[1] Hollingsworth RE, Jansen K (2019). Turning the corner on therapeutic cancer vaccines. NPJ Vaccines.

[2] Saxena M (2021). Therapeutic cancer vaccines. Nat Rev Cancer.

[3] Ott PA (2017). An immunogenic personal neoantigen vaccine for patients with melanoma. Nature.

[4] Sahin U (2017). Personalized RNA mutanome vaccines mobilize poly-specific therapeutic immunity against cancer. Nature.

[5] Sahin U (2020). An RNA vaccine drives immunity in checkpoint-inhibitor-treated melanoma. Nature.

[6] Ott PA (2020). A phase Ib trial of personalized neoantigen therapy plus anti-PD-1 in patients with advanced melanoma, non-small cell lung cancer, or bladder cancer. Cell.

[7] Zaidi N (2020). Can personalized neoantigens raise the T cell bar?. Cell.

[8] Hilf N (2019). Actively personalized vaccination trial for newly diagnosed glioblastoma. Nature.

[9] Keskin DB (2019). Neoantigen vaccine generates intratumoral T cell responses in phase Ib glioblastoma trial. Nature.

[10] Zaidi N (2020). Role of in silico structural modeling in predicting immunogenic neoepitopes for cancer vaccine development. JCI Insight.

[11] Chen Z (2021). A neoantigen-based peptide vaccine for patients with advanced pancreatic cancer refractory to standard treatment. Front Immunol.

[12] Malone RW (1989). Cationic liposome-mediated RNA transfection. Proc Natl Acad Sci U S A.

[13] Wolff JA (1990). Direct gene transfer into mouse muscle in vivo. Science.

[14] Martinon F (1993). Induction of virus-specific cytotoxic T lymphocytes in vivo by liposome-entrapped mRNA. Eur J Immunol.

[15] Conry RM (1995). Characterization of a messenger RNA polynucleotide vaccine vector. Cancer Res.

[16] Conry RM (1995). A carcinoembryonic antigen polynucleotide vaccine for human clinical use. Cancer Gene Ther.

[17] Conry RM (1995). A carcinoembryonic antigen polynucleotide vaccine has in vivo antitumor activity. Gene Ther.

[18] Strong TV (1997). Incorporation of beta-globin untranslated regions into a Sindbis virus vector for augmentation of heterologous mRNA expression. Gene Ther.

[19] Atkins GJ (1996). Manipulation of the Semliki Forest virus genome and its potential for vaccine construction. Mol Biotechnol.

[20] Ying H (1999). Cancer therapy using a self-replicating RNA vaccine. Nat Med.

[21] Zhou WZ (1999). RNA melanoma vaccine: induction of antitumor immunity by human glycoprotein 100 mRNA immunization. Hum Gene Ther.

[22] Boczkowski D (1996). Dendritic cells pulsed with RNA are potent antigen-presenting cells in vitro and in vivo. J Exp Med.

[23] Nair SK (1998). Immunotherapy of cancer with dendritic cell-based vaccines. Gene Ther.

[24] Nair SK (1998). Induction of primary carcinoembryonic antigen (CEA)-specific cytotoxic T lymphocytes in vitro using human dendritic cells transfected with RNA. Nat Biotechnol.

[25] Ashley DM (1997). Bone marrow-generated dendritic cells pulsed with tumor extracts or tumor RNA induce antitumor immunity against central nervous system tumors. J Exp Med.

[26] Chahal JS (2016). Dendrimer-RNA nanoparticles generate protective immunity against lethal Ebola, H1N1 influenza, and Toxoplasma gondii challenges with a single dose. Proc Natl Acad Sci U S A.

[27] Bahl K (2017). Preclinical and clinical demonstration of immunogenicity by mRNA vaccines against H10N8 and H7N9 influenza viruses. Mol Ther.

[28] Pardi N (2017). Administration of nucleoside-modified mRNA encoding broadly neutralizing antibody protects humanized mice from HIV-1 challenge. Nat Commun.

[29] Pardi N (2017). Zika virus protection by a single low-dose nucleoside-modified mRNA vaccination. Nature.

[30] Schnee M (2016). An mRNA vaccine encoding rabies virus glycoprotein induces protection against lethal infection in mice and correlates of protection in adult and newborn pigs. PLoS Negl Trop Dis.

[31] Baden LR (2021). Efficacy and safety of the mRNA-1273 SARS-CoV-2 Vaccine. N Engl J Med.

[32] Polack FP (2020). Safety and efficacy of the BNT162b2 mRNA Covid-19 vaccine. N Engl J Med.

[33] Sahin U (2021). BNT162b2 vaccine induces neutralizing antibodies and poly-specific T cells in humans. Nature.

[34] Anderson EJ (2020). Safety and immunogenicity of SARS-CoV-2 mRNA-1273 vaccine in older adults. N Engl J Med.

[35] Goel RR (2021). mRNA vaccines induce durable immune memory to SARS-CoV-2 and variants of concern. Science.

[36] Jackson LA (2020). An mRNA vaccine against SARS-CoV-2 - preliminary report. N Engl J Med.

[37] Sahin U (2020). COVID-19 vaccine BNT162b1 elicits human antibody and T_H_1 T cell responses. Nature.

[38] Oberhardt V (2021). Rapid and stable mobilization of CD8^+^ T cells by SARS-CoV-2 mRNA vaccine. Nature.

[39] Mateus J (2021). Low-dose mRNA-1273 COVID-19 vaccine generates durable memory enhanced by cross-reactive T cells. Science.

[40] Corbett KS (2020). Evaluation of the mRNA-1273 Vaccine against SARS-CoV-2 in nonhuman primates. N Engl J Med.

[42] Holm MR, Poland GA (2021). Critical aspects of packaging, storage, preparation, and administration of mRNA and adenovirus-vectored COVID-19 vaccines for optimal efficacy. Vaccine.

[43] Zhao P (2020). Long-term storage of lipid-like nanoparticles for mRNA delivery. Bioact Mater.

[44] Crommelin DJA (2021). Addressing the cold reality of mRNA vaccine stability. J Pharm Sci.

[45] Hou X (2021). Lipid nanoparticles for mRNA delivery. Nat Rev Mater.

[46] Meng C (2021). Nanoplatforms for mRNA therapeutics. Advanced Therapeutics.

[47] Kim D (2021). Advances in vaccine delivery systems against viral infectious diseases. Drug Deliv Transl Res.

[48] Walsh EE (2020). Safety and immunogenicity of two RNA-based covid-19 vaccine candidates. N Engl J Med.

[49] Ali K (2021). Evaluation of mRNA-1273 SARS-CoV-2 vaccine in adolescents. N Engl J Med.

[50] Kelso JM (2021). Anaphylactic reactions to novel mRNA SARS-CoV-2/COVID-19 vaccines. Vaccine.

[51] Sellaturay P (2021). Polyethylene glycol-induced systemic allergic reactions (anaphylaxis). J Allergy Clin Immunol Pract.

[52] Shimabukuro TT (2021). Reports of anaphylaxis after receipt of mRNA COVID-19 vaccines in the US—December 14, 2020–January 18, 2021. JAMA.

[53] Pollard AJ, Bijker EM (2021). A guide to vaccinology: from basic principles to new developments. Nat Rev Immunol.

[54] Ni L (2020). Detection of SARS-CoV-2-specific humoral and cellular immunity in COVID-19 convalescent individuals. Immunity.

[55] Wen W (2020). Immune cell profiling of COVID-19 patients in the recovery stage by single-cell sequencing. Cell Discov.

[56] Liao M (2020). Single-cell landscape of bronchoalveolar immune cells in patients with COVID-19. Nat Med.

[57] Valpione S (2021). The T cell receptor repertoire of tumor infiltrating T cells is predictive and prognostic for cancer survival. Nat Commun.

[58] Kreiter S (2015). Mutant MHC class II epitopes drive therapeutic immune responses to cancer. Nature.

[59] Linnemann C (2015). High-throughput epitope discovery reveals frequent recognition of neo-antigens by CD4^+^ T cells in human melanoma. Nat Med.

[60] Alspach E (2019). MHC-II neoantigens shape tumour immunity and response to immunotherapy. Nature.

[61] Quezada SA (2010). Tumor-reactive CD4(+) T cells develop cytotoxic activity and eradicate large established melanoma after transfer into lymphopenic hosts. J Exp Med.

[62] Spitzer MH (2017). Systemic immunity is required for effective cancer immunotherapy. Cell.

[63] Xie Y (2010). Naive tumor-specific CD4(+) T cells differentiated in vivo eradicate established melanoma. J Exp Med.

[64] Zhang L (2018). Lineage tracking reveals dynamic relationships of T cells in colorectal cancer. Nature.

[65] Diken M (2011). Selective uptake of naked vaccine RNA by dendritic cells is driven by macropinocytosis and abrogated upon DC maturation. Gene Ther.

[66] Binnewies M (2018). Understanding the tumor immune microenvironment (TIME) for effective therapy. Nat Med.

[67] Li Y (2020). Multifunctional oncolytic nanoparticles deliver self-replicating IL-12 RNA to eliminate established tumors and prime systemic immunity. Nat Cancer.

[68] Pardi N (2015). Expression kinetics of nucleoside-modified mRNA delivered in lipid nanoparticles to mice by various routes. J Control Release.

[69] Leyman B (2018). Comparison of the expression kinetics and immunostimulatory activity of replicating mRNA, nonreplicating mRNA, and pDNA after intradermal electroporation in pigs. Mol Pharm.

[70] Beissert T (2020). A Trans-amplifying RNA vaccine strategy for induction of potent protective immunity. Mol Ther.

[71] Blakney AK (2018). Structural components for amplification of positive and negative strand VEEV splitzicons. Front Mol Biosci.

[72] Spuul P (2011). Assembly of alphavirus replication complexes from RNA and protein components in a novel trans-replication system in mammalian cells. J Virol.

[73] Heil F (2004). Species-specific recognition of single-stranded RNA via toll-like receptor 7 and 8. Science.

[74] Karikó K (2008). Incorporation of pseudouridine into mRNA yields superior nonimmunogenic vector with increased translational capacity and biological stability. Mol Ther.

[75] Pardi N, Weissman D (2017). Nucleoside modified mRNA vaccines for infectious diseases. Methods Mol Biol.

[76] Andries O (2015). N(1)-methylpseudouridine-incorporated mRNA outperforms pseudouridine-incorporated mRNA by providing enhanced protein expression and reduced immunogenicity in mammalian cell lines and mice. J Control Release.

[77] Karikó K (2005). Suppression of RNA recognition by Toll-like receptors: the impact of nucleoside modification and the evolutionary origin of RNA. Immunity.

[78] Thess A (2015). Sequence-engineered mRNA without chemical nucleoside modifications enables an effective protein therapy in large animals. Mol Ther.

[79] Baiersdörfer M (2019). A facile method for the removal of dsRNA contaminant from in vitro-transcribed mRNA. Mol Ther Nucleic Acids.

[80] Karikó K (2011). Generating the optimal mRNA for therapy: HPLC purification eliminates immune activation and improves translation of nucleoside-modified, protein-encoding mRNA. Nucleic Acids Res.

[81] Kim I (2007). Rapid purification of RNAs using fast performance liquid chromatography (FPLC). RNA.

[82] Miao L (2019). Delivery of mRNA vaccines with heterocyclic lipids increases anti-tumor efficacy by STING-mediated immune cell activation. Nat Biotechnol.

[83] Tse SW (2021). mRNA-encoded, constitutively active STING. Mol Ther.

[84] De Beuckelaer A (2017). Type I interferons modulate CD8. Trends Mol Med.

[85] Furuichi Y (2015). Discovery of m(7)G-cap in eukaryotic mRNAs. Proc Jpn Acad Ser B Phys Biol Sci.

[86] Ramanathan A (2016). mRNA capping: biological functions and applications. Nucleic Acids Res.

[87] Leung DW, Amarasinghe GK (2016). When your cap matters: structural insights into self vs non-self recognition of 5′ RNA by immunomodulatory host proteins. Curr Opin Struct Biol.

[88] Gebhardt A (2017). Discrimination of self and non-self ribonucleic acids. J Interferon Cytokine Res.

[89] Grudzien-Nogalska E (2007). Phosphorothioate cap analogs stabilize mRNA and increase translational efficiency in mammalian cells. RNA.

[90] Grudzien-Nogalska E (2007). Synthesis of anti-reverse cap analogs (ARCAs) and their applications in mRNA translation and stability. Methods Enzymol.

[91] Jemielity J (2003). Novel “anti-reverse” cap analogs with superior translational properties. RNA.

[92] Grudzien-Nogalska E, Kiledjian M (2017). New insights into decapping enzymes and selective mRNA decay. Wiley Interdiscip Rev RNA.

[93] Topisirovic I (2011). Cap and cap-binding proteins in the control of gene expression. Wiley Interdiscip Rev RNA.

[94] Grudzien E (2006). Differential inhibition of mRNA degradation pathways by novel cap analogs. J Biol Chem.

[95] Kuhn AN (2010). Phosphorothioate cap analogs increase stability and translational efficiency of RNA vaccines in immature dendritic cells and induce superior immune responses in vivo. Gene Ther.

[96] Strenkowska M (2016). Cap analogs modified with 1,2-dithiodiphosphate moiety protect mRNA from decapping and enhance its translational potential. Nucleic Acids Res.

[97] Su W (2011). Translation, stability, and resistance to decapping of mRNAs containing caps substituted in the triphosphate chain with BH3, Se, and NH. RNA.

[98] Ziemniak M (2013). Synthesis and evaluation of fluorescent cap analogues for mRNA labelling. RSC Adv.

[99] Niedzwiecka A (2002). Biophysical studies of eIF4E cap-binding protein: recognition of mRNA 5′ cap structure and synthetic fragments of eIF4G and 4E-BP1 proteins. J Mol Biol.

[100] Worch R (2005). Specificity of recognition of mRNA 5′ cap by human nuclear cap-binding complex. RNA.

[101] van Dülmen M (2021). Chemo-enzymatic modification of the 5′ cap maintains translation and increases immunogenic properties of mRNA. Angew Chem Int Ed Engl.

[102] Sonenberg N, Hinnebusch AG (2009). Regulation of translation initiation in eukaryotes: mechanisms and biological targets. Cell.

[103] Mugridge JS (2018). Structural and molecular mechanisms for the control of eukaryotic 5′-3′ mRNA decay. Nat Struct Mol Biol.

[104] Leppek K (2018). Functional 5′ UTR mRNA structures in eukaryotic translation regulation and how to find them. Nat Rev Mol Cell Biol.

[105] Chen SJ (2008). RNA folding: conformational statistics, folding kinetics, and ion electrostatics. Annu Rev Biophys.

[106] Pelletier J, Sonenberg N (1985). Insertion mutagenesis to increase secondary structure within the 5′ noncoding region of a eukaryotic mRNA reduces translational efficiency. Cell.

[107] Vagner S (2001). Irresistible IRES. Attracting the translation machinery to internal ribosome entry sites. EMBO Rep.

[108] Kozak M (1987). At least six nucleotides preceding the AUG initiator codon enhance translation in mammalian cells. J Mol Biol.

[109] Sample PJ (2019). Human 5′ UTR design and variant effect prediction from a massively parallel translation assay. Nat Biotechnol.

[110] Schwanhäusser B (2011). Global quantification of mammalian gene expression control. Nature.

[111] Kwon H (2018). Emergence of synthetic mRNA: in vitro synthesis of mRNA and its applications in regenerative medicine. Biomaterials.

[112] Linares-Fernández S (2020). Tailoring mRNA vaccine to balance innate/adaptive immune response. Trends Mol Med.

[113] Holtkamp S (2006). Modification of antigen-encoding RNA increases stability, translational efficacy, and T-cell stimulatory capacity of dendritic cells. Blood.

[114] Hu W (2009). Co-translational mRNA decay in Saccharomyces cerevisiae. Nature.

[115] Presnyak V (2015). Codon optimality is a major determinant of mRNA stability. Cell.

[116] Rivas E (2020). RNA structure prediction using positive and negative evolutionary information. PLoS Comput Biol.

[117] Zhang H (2019). A new method of RNA secondary structure prediction based on convolutional neural network and dynamic programming. Front Genet.

[118] Leppek K Combinatorial optimization of mRNA structure, stability, and translation for RNA-based therapeutics. bioRxiv.

[119] Goodarzi H (2016). Modulated expression of specific tRNAs drives gene expression and cancer progression. Cell.

[120] Dittmar KA (2006). Tissue-specific differences in human transfer RNA expression. PLoS Genet.

[121] Strzelecka D (2020). Phosphodiester modifications in mRNA poly(A) tail prevent deadenylation without compromising protein expression. RNA.

[122] Anhäuser L (2019). Multiple covalent fluorescence labeling of eukaryotic mRNA at the poly(A) tail enhances translation and can be performed in living cells. Nucleic Acids Res.

[123] Westerich KJ (2020). Bioorthogonal mRNA labeling at the poly(A) tail for imaging localization and dynamics in live zebrafish embryos. Chem Sci.

[124] Chang JT (2014). Molecular regulation of effector and memory T cell differentiation. Nat Immunol.

[125] Mami-Chouaib F (2018). Resident memory T cells, critical components in tumor immunology. J Immunother Cancer.

[126] Abbasi S, Uchida S (2021). Multifunctional immunoadjuvants for use in minimalist nucleic acid vaccines. Pharmaceutics.

[127] Yang J (2019). Hybrid nanovaccine for the co-delivery of the mRNA antigen and adjuvant. Nanoscale.

[128] Bontkes HJ (2007). Dendritic cells transfected with interleukin-12 and tumor-associated antigen messenger RNA induce high avidity cytotoxic T cells. Gene Ther.

[129] Naka T (2008). Tumor vaccine therapy against recrudescent tumor using dendritic cells simultaneously transfected with tumor RNA and granulocyte macrophage colony-stimulating factor RNA. Cancer Sci.

[130] Van den Bergh J (2015). Transpresentation of interleukin-15 by IL-15/IL-15Rα mRNA-engineered human dendritic cells boosts antitumoral natural killer cell activity. Oncotarget.

[131] Van den Bergh JMJ (2017). Characterization of interleukin-15-transpresenting dendritic cells for clinical use. J Immunol Res.

[132] Hotz C (2021). Local delivery of mRNA-encoded cytokines promotes antitumor immunity and tumor eradication across multiple preclinical tumor models. Sci Transl Med.

[133] Bialkowski L (2016). Intralymphatic mRNA vaccine induces CD8 T-cell responses that inhibit the growth of mucosally located tumours. Sci Rep.

[134] De Keersmaecker B (2020). TriMix and tumor antigen mRNA electroporated dendritic cell vaccination plus ipilimumab: link between T-cell activation and clinical responses in advanced melanoma. J Immunother Cancer.

[135] Liu L (2018). Combination immunotherapy of MUC1 mRNA Nano-vaccine and CTLA-4 blockade effectively inhibits growth of triple negative breast cancer. Mol Ther.

[136] Wang Y (2018). mRNA vaccine with antigen-specific checkpoint blockade induces an enhanced immune response against established melanoma. Mol Ther.

[137] Huo M (2017). Tumor-targeted delivery of sunitinib base enhances vaccine therapy for advanced melanoma by remodeling the tumor microenvironment. J Control Release.

[138] Reinhard K (2020). An RNA vaccine drives expansion and efficacy of claudin-CAR-T cells against solid tumors. Science.

[139] Billingsley MM (2020). Ionizable lipid nanoparticle-mediated mRNA delivery for human CAR T cell engineering. Nano Lett.

[140] Kauffman KJ (2016). Materials for non-viral intracellular delivery of messenger RNA therapeutics. J Control Release.

[141] Guan S, Rosenecker J (2017). Nanotechnologies in delivery of mRNA therapeutics using nonviral vector-based delivery systems. Gene Ther.

[142] Breton G (2015). Defining human dendritic cell progenitors by multiparametric flow cytometry. Nat Protoc.

[143] Merad M (2013). The dendritic cell lineage: ontogeny and function of dendritic cells and their subsets in the steady state and the inflamed setting. Annu Rev Immunol.

[144] Spranger S (2010). Generation of Th1-polarizing dendritic cells using the TLR7/8 agonist CL075. J Immunol.

[145] Islam MA (2018). Restoration of tumour-growth suppression in vivo via systemic nanoparticle-mediated delivery of PTEN mRNA. Nat Biomed Eng.

[146] Phua KK (2013). Transfection efficiency and transgene expression kinetics of mRNA delivered in naked and nanoparticle format. J Control Release.

[147] Phua KK (2014). Whole blood cells loaded with messenger RNA as an anti-tumor vaccine. Adv Healthc Mater.

[148] Gupta A (2021). Nucleic acid delivery for therapeutic applications. Adv Drug Deliv Rev.

[149] Liu T (2021). Development and delivery systems of mRNA vaccines. Front Bioeng Biotechnol.

[150] Jindal AB (2017). The effect of particle shape on cellular interaction and drug delivery applications of micro- and nanoparticles. Int J Pharm.

[151] Nakamura T, Harashima H (2020). Dawn of lipid nanoparticles in lymph node targeting: Potential in cancer immunotherapy. Adv Drug Deliv Rev.

[152] Kranz LM (2016). Systemic RNA delivery to dendritic cells exploits antiviral defence for cancer immunotherapy. Nature.

[153] Manolova V (2008). Nanoparticles target distinct dendritic cell populations according to their size. Eur J Immunol.

[154] Reddy ST (2006). In vivo targeting of dendritic cells in lymph nodes with poly(propylene sulfide) nanoparticles. J Control Release.

[155] Reddy ST (2007). Exploiting lymphatic transport and complement activation in nanoparticle vaccines. Nat Biotechnol.

[156] Markov OV (2015). Multicomponent mannose-containing liposomes efficiently deliver RNA in murine immature dendritic cells and provide productive anti-tumour response in murine melanoma model. J Control Release.

[157] Salah A (2021). Insights into dendritic cells in cancer immunotherapy: from bench to clinical applications. Front Cell Dev Biol.

[158] Abbas A (2020). The activation trajectory of plasmacytoid dendritic cells in vivo during a viral infection. Nat Immunol.

[159] Bosteels C, Scott CL (2020). Transcriptional regulation of DC fate specification. Mol Immunol.

[160] Yin Y (2021). In situ transforming RNA nanovaccines from polyethylenimine functionalized graphene oxide hydrogel for durable cancer immunotherapy. Nano Lett.

[161] https://www.fda.gov/media/144412/download.

[162] https://www.modernatx.com/sites/default/files/mRNA-1273-P301-Protocol.pdf.

[163] Li B (2015). An orthogonal array optimization of lipid-like nanoparticles for mRNA delivery in vivo. Nano Lett.

[164] Li Y (2019). In vitro evolution of enhanced RNA replicons for immunotherapy. Sci Rep.

[165] Kong N (2019). Synthetic mRNA nanoparticle-mediated restoration of p53 tumor suppressor sensitizes p53-deficient cancers to mTOR inhibition. Sci Transl Med.

[166] Cheng Q (2020). Selective organ targeting (SORT) nanoparticles for tissue-specific mRNA delivery and CRISPR-Cas gene editing. Nat Nanotechnol.

[167] Sabnis S (2018). A novel amino lipid series for mRNA delivery: improved endosomal escape and sustained pharmacology and safety in non-human primates. Mol Ther.

[168] Patel S (2017). Boosting intracellular delivery of lipid nanoparticle-encapsulated mRNA. Nano Lett.

[169] Jiang Y (2020). Quantitating endosomal escape of a library of polymers for mRNA delivery. Nano Lett.

[170] Patel SG (2019). Cell-penetrating peptide sequence and modification dependent uptake and subcellular distribution of green florescent protein in different cell lines. Sci Rep.

[171] Hassett KJ (2019). Optimization of lipid nanoparticles for intramuscular administration of mRNA vaccines. Mol Ther Nucleic Acids.

[172] Clausen BE, Stoitzner P (2015). Functional specialization of skin dendritic cell subsets in regulating T cell responses. Front Immunol.

[173] Honda T (2019). Antigen presentation and adaptive immune responses in skin. Int Immunol.

[174] Ols S, Yang L (2020). Route of vaccine administration alters antigen trafficking but not innate or adaptive immunity. Cell Rep.

[175] Sayour EJ (2017). Systemic activation of antigen-presenting cells via RNA-loaded nanoparticles. Oncoimmunology.

[176] Chung DJ (2017). Langerhans-type dendritic cells electroporated with TRP-2 mRNA stimulate cellular immunity against melanoma: results of a phase I vaccine trial. Oncoimmunology.

[177] Cafri G (2020). mRNA vaccine-induced neoantigen-specific T cell immunity in patients with gastrointestinal cancer. J Clin Invest.

[178] Van Lint S (2012). Preclinical evaluation of TriMix and antigen mRNA-based antitumor therapy. Cancer Res.

[179] Wilgenhof S (2013). A phase IB study on intravenous synthetic mRNA electroporated dendritic cell immunotherapy in pretreated advanced melanoma patients. Ann Oncol.

[180] Jansen Y (2020). A randomized controlled phase II clinical trial on mRNA electroporated autologous monocyte-derived dendritic cells (TriMixDC-MEL) as adjuvant treatment for stage III/IV melanoma patients who are disease-free following the resection of macrometastases. Cancer Immunol Immunother.

[181] Van Tendeloo VF (2010). Induction of complete and molecular remissions in acute myeloid leukemia by Wilms’ tumor 1 antigen-targeted dendritic cell vaccination. Proc Natl Acad Sci U S A.

[182] Amin A (2015). Survival with AGS-003, an autologous dendritic cell-based immunotherapy, in combination with sunitinib in unfavorable risk patients with advanced renal cell carcinoma (rcc): phase 2 study results. J Immunother Cancer.

[183] Kyte JA (2016). Immune response and long-term clinical outcome in advanced melanoma patients vaccinated with tumor-mRNA-transfected dendritic cells. Oncoimmunology.

[184] Fernandez MA (2019). A phase I study (E011-MEL) of a TriMix-based mRNA immunotherapy (ECI-006) in resected melanoma patients: Analysis of safety and immunogenicity. J Clin Oncol.

[185] Rittig SM (2016). Long-term survival correlates with immunological responses in renal cell carcinoma patients treated with mRNA-based immunotherapy. Oncoimmunology.

[186] Sebastian M (2019). A phase I/IIa study of the mRNA-based cancer immunotherapy CV9201 in patients with stage IIIB/IV non-small cell lung cancer. Cancer Immunol Immunother.

[187] Kübler H (2015). Self-adjuvanted mRNA vaccination in advanced prostate cancer patients: a first-in-man phase I/IIa study. J Immunother Cancer.

[189] Patel MR (2020). A phase I study of mRNA-2752, a lipid nanoparticle encapsulating mRNAs encoding human OX40L, IL-23, and IL-36γ, for intratumoral (iTu) injection alone and in combination with durvalumab. J Clin Oncol.

[190] https://clinicaltrials.gov.NCT03313778.

[191] Burris HA (2019). A phase I multicenter study to assess the safety, tolerability, and immunogenicity of mRNA-4157 alone in patients with resected solid tumors and in combination with pembrolizumab in patients with unresectable solid tumors. J Clin Oncol.

